# AbDb: antibody structure database—a database of PDB-derived antibody structures

**DOI:** 10.1093/database/bay040

**Published:** 2018-04-27

**Authors:** Saba Ferdous, Andrew C R Martin

**Affiliations:** Division of Biosciences, Institute of Structural and Molecular Biology, University College London, Darwin Building, Gower Street, London WC1E 6BT, UK

## Abstract

In order to analyse structures of proteins of a particular class, these need to be extracted from Protein Data Bank (PDB) files. In the case of antibodies, there are a number of special considerations: (i) identifying antibodies in the PDB is not trivial, (ii) they may be crystallized with or without antigen, (iii) for analysis purposes, one is normally only interested in the *Fv* region of the antibody, (iv) structural analysis of epitopes, in particular, requires individual antibody–antigen complexes from a PDB file which may contain multiple copies of the same, or different, antibodies and (v) standard numbering schemes should be applied. Consequently, there is a need for a specialist resource containing pre-numbered non-redundant antibody *Fv* structures with their cognate antigens. We have created an automatically updated resource, AbDb, which collects the *Fv* regions from antibody structures using information from our SACS database which summarizes antibody structures from the PDB. PDB files containing multiple structures are split and numbered and each antibody structure is associated with its antigen where available. Antibody structures with only light or heavy chains have also been processed and sequences of antibodies are compared to identify multiple structures of the same antibody. The data may be queried on the basis of PDB code, or the name or species of the antibody or antigen, and the complete datasets may be downloaded.

**Database URL**: www.bioinf.org.uk/abs/abdb/

## Introduction

As well as their role in the immune system, since an antibody was first administered to a human as a drug in 1986, the field of therapeutic antibody development has grown enormously. Antibody therapeutics now represent one third of drugs in development, being used in a range of diseases from infection to cancer (1). Antibodies are capable of binding with high specificity and affinity and, unlike small molecule drugs, are able to bind large flat surfaces rather than pockets. Their importance in biomedical research, diagnostics and therapeutics (2) has led to a need to understand the basis of antibody specificity, binding affinity, stability and structure. A number of databases contain antibody sequence data (3–9), while others provide summaries of, or access to, structural data (6, 7, 9, 10, 11). In the Protein Data Bank (PDB), managed by the Worldwide PDB (wwPDB) (12) antibody structures currently represent ∼2.1% of entries (January 2018).

There are a number of resources that make information about antibody structures available. The first of these was SACS (10) which simply provides a regularly-updated list of antibody structures from the PDB with some basic annotations. abYsis (6) is predominantly an analysis resource; it brings together antibody sequence data from different publicly available data sources including Kabat, EMBL-ENA, the PDB and, optionally, IMGT, providing tools to analyse antibodies within the web-based resource (e.g. numbering (13), canonicals (14, 15), unusual residues, humanness (16), germline source, etc.). IMGT/3Dstructure-DB (7) provides an interface for the inspection of antibody structure data including bound protein antigens. SAbDab (11) provides a web-based search interface and allows the original antibody PDB files to be downloaded, as well as PDB files with Chothia numbering applied. These numbered files also contain information about the pairing of light and heavy chains and identify associated antigen chains.

**Figure 1.**
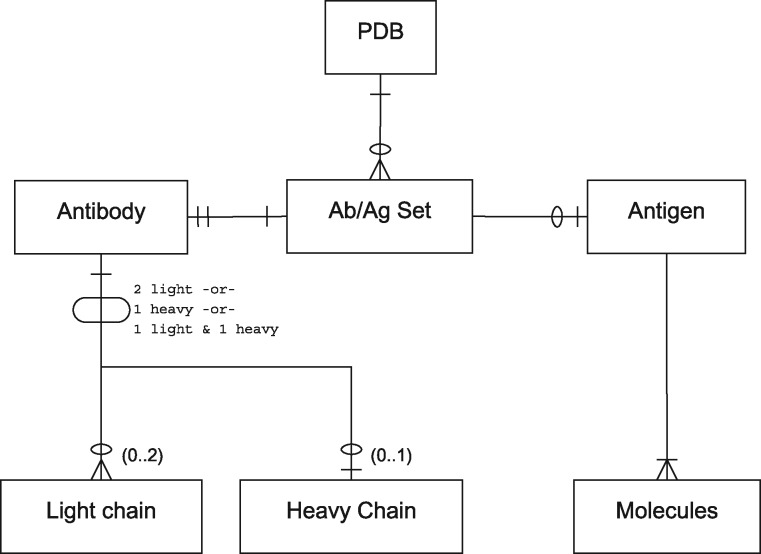
** **A PDB file may have single or multiple antibody–antigen (Ab/Ag) sets. The notation is in Information Engineering Style: a single line indicates one item; two lines indicates exactly one mandatory item; a single line and a circle represents zero or one item; a crowsfoot with a circle indicates zero or more items; a crowsfoot with a line indicates one or more mandatory items. Each Ab/Ag Set contains exactly one antibody. An antibody may be ‘complete’ (containing one light and one heavy chain), or may be a light-chain dimer or heavy-chain monomer. Each Ab/Ag Set also contains zero or one antigen which contains one or more molecules or chains. Note that the same antigen may take part in more than one Ab/Ag Set.

For the purposes of this paper, we use the term ‘antibody’ to refer to antibody-derived antigen binding fragments including light-chain dimers and camelid VHHs (isolated variable heavy chains). Thus the relationship between antibody and antigen chains in a PDB file is complex. Antibodies may consist of a light-chain dimer, a single heavy chain or a conventional light/heavy complex. Likewise, antigens may consist of small molecules or of one or more protein or non-protein chains and the epitope to which an antibody binds may span more than one chain. A single PDB file may contain multiple copies of the same antibody or multiple antibodies bound to different parts of the same antigen. Figure 1 provides an entity-relationship (ER) diagram describing these scenarios.

Antigens having multiple chains have proved a particular challenge for the analysis of discontinuous epitopes that span multiple antigen chains and some studies have simply excluded such examples from the analysis (17). Another problem with antibody structure related resources is the incorrect identification of ‘antibody-binding proteins’ as antigens. As a result, epitopes and paratopes, such as those identified by IMGT/3Dstructure-DB, can be incorrect. For example, PDB file 1DEE (18), contains a complex between the *Fab* fragment (antigen-binding fragment) of IgM antibody 2A2 and Ig-binding domain D of the *Staphylococcus aureus* virulence factor, protein A (SpA). SpA binds to framework residues of the V_H_ (heavy variable) domain that are highly conserved in human VH3 germline derived domains; the complementarity determining regions (CDRs) are not involved. Both IMGT/3Dstructure-DB and SAbDab erroneously indicate that this antibody is in a complex with antigen. In addition, antibodies themselves can act as antigens; other antibodies can bind to the framework, or so-called anti-idiotypic antibodies can bind the CDRs. When an antibody binds to the framework there is a clear differentiation between antibody and antigen. In the case of anti-idiotypic complexes, without additional information, it is impossible to determine which is truly the antibody and which is the antigen.

We set out to develop an automatically maintained resource to address these and other problems. In particular we wished our resource to:

- Correctly identify antibody-binding proteins and not treat them as antigens,
- Provide downloadable isolated *Fv* (variable) fragments discarding constant domains,
- Provide multiple numbering schemes [Kabat (19), Chothia (20) and Martin (13)],
- Split PDB files containing multiple antibodies into separate files, each containing antigen chains as appropriate. For example, PDB file 4FQR contains 12 copies of the same antibody and these need to be split into separate files; in 3ULU, three different antibodies are bound to the same antigen chain so this must be replicated between the three files,
- Correctly handle cases where the antibody binds multiple chains of an antigen (e.g. 2FEE, 3PJS, 3ZTN),
- Deal with instances of anti-idiotypic antibodies where each antibody is treated both as antibody and as antigen,
- Provide information on redundancy (representative antibodies and lists of non-redundant antibodies and redundant sets),
- Provide information on antibodies that are available both bound and unbound,
- Provide separate datasets for antibody–antigen complexes where the antigen is a protein and where it is a non-protein (e.g. PDB code 4M7J binds carbohydrate, 1A0Q and 1BAF bind hapten, 3VW3 binds DNA and 2R8S binds RNA),
- Correctly handle light-chain-only antibodies (such as Bence-Jones dimers) and heavy-chain-only (VHH) antibodies,
- Provide a dataset that is as clean and robust as possible, potentially at the expense of excluding a small percentage of the available structures,
- Provide a web interface that can be used to search by PDB code, antibody or antigen name or species, but most importantly allows the download of complete datasets of free antibodies, antibodies complexed with protein antigens or antibodies complexed with non-protein antigens, as well as lists of redundant antibodies and complexed/uncomplexed pairs. In addition, we wished to separate ‘complete’ antibodies (i.e. paired light and heavy chains), light-chain dimers and heavy-chain-only structures.

To our knowledge, none of the other available resources deals with all these problems.

## Materials and Methods

### Data processing pipeline

PDB data are mirrored locally and automatically uncompressed using *ftpmirror* which may be obtained from github.com/AndrewCRMartin/bioscripts. Identification of antibody structures in the PDB is not trivial because they may be described as ‘antibody’ or ‘immunoglobulin’ and both keywords may be used in other contexts; without further precautions, simple homology searches will also identify related molecules such as T-cell receptors. Identification of antibody PDB files is handled by an enhanced version of the SACS procedure described previously (10). SACS data were obtained as an XML file from www.bioinf.org.uk/abs/sacs/ and parsed to obtain a list of PDB codes.

**Figure 2.**
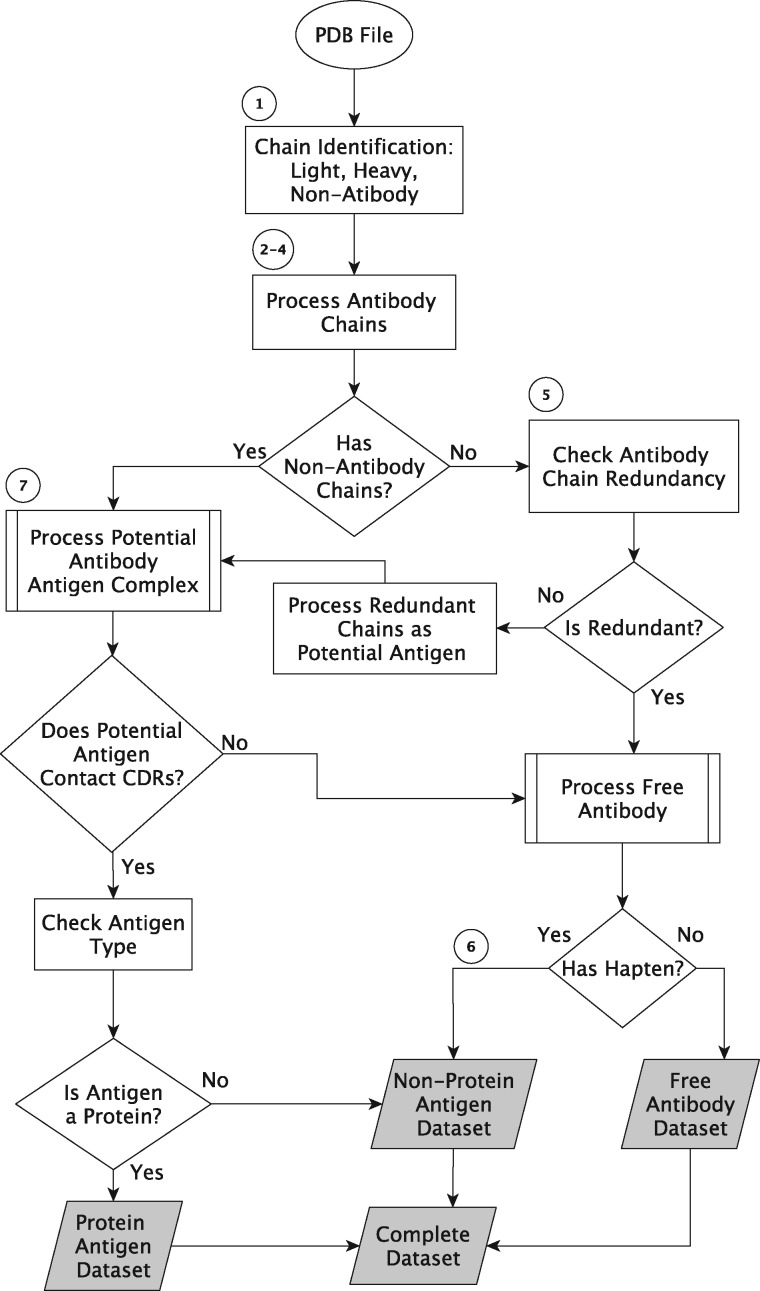
Data processing algorithm outline. The circled numbers refer to steps in the text and the shaded boxes are the output datasets.

Figure 2 shows a work flow diagram, for which the major processing steps are explained in detail below.

**Step 1: Identify Chain Types.** Each PDB file is processed to identify the chain types present: light, heavy or antigen. An in-house program, *idabchain*, also used by SACS (10), aligns the sequence of each PDB chain with consensus light- and heavy-chain variable domain sequences. Each chain is provisionally assigned as light or heavy depending which scores higher and the highest score is recorded. Each chain that was provisionally identified as light (respectively heavy) in the previous step is then reassigned as antigen if it does not have the highest score for a match against the light (respectively heavy) chain sequence (i.e. other chains score more highly) and its score is <80%. If SEQRES records are available, then the sequence is read from both the SEQRES and ATOM records and the higher score is selected—this deals with missing residues in ATOM records and also cases where the SEQRES records contain the leader sequence the signal sequence present at the N-terminus of immature antibodies.

**Step 2: Assign antibody type.** A decision is then made as to whether the antibody is ‘complete’ (containing both light and heavy chains), light-chain only or heavy-chain only. Complete antibodies and antibodies containing a single chain type are then processed differently to deal with correct chain pairing. In particular, for some cases of light-chain-only antibodies, the symmetry of the two light chains means that only one chain appears in the original PDB file. *pdbsymm* from BiopTools (21) exploits the REMARK 350 BIOMT records to construct a new PDB file containing the two copies of the light chain.

**Step 3: Splitting and Numbering.** In a PDB file, peptide, protein, DNA and RNA antigens are present as ATOM records with distinct chain labels whereas lipid, carbohydrate and hapten antigens are represented by HETATM records and may have the same chain label as one of the protein chains. The file is then split such that each chain (as defined by the chain label in the PDB file) is placed in its own file and associated HETATMs are removed to be handled later. The files containing the antibody chains are then numbered according to each of the three numbering schemes (Kabat, Chothia and Martin) using *Abnum* (13) and constant domains are discarded.

**Figure 3.**
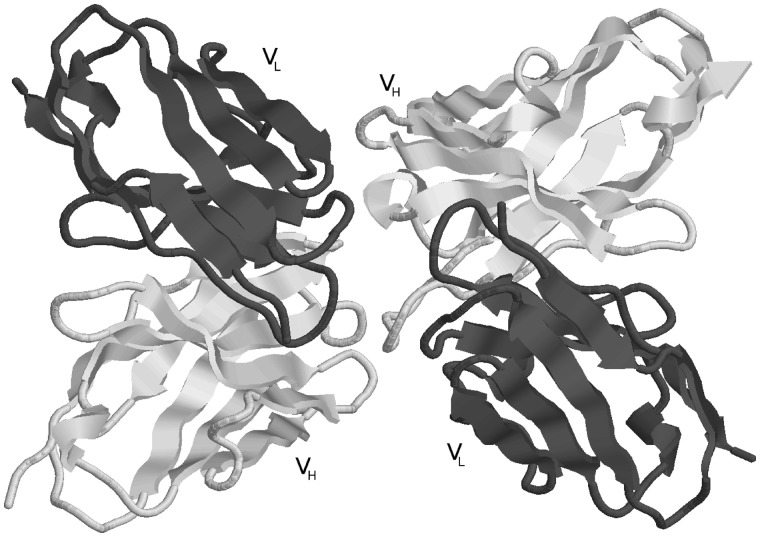
An example of an anti-idiotype antibody where one antibody is interacting with the CDRs of another antibody. The processing pipeline generates two structures with each antibody treated as antibody and antigen, respectively (PDB entry: 1DVF).

**Step 4: Antibody Reassembly.** Most PDB files containing ‘complete’ antibodies have either duplets of light and heavy chains, or triplets of light, heavy and antigen chains which appear in that order (i.e. light, heavy or light, heavy, antigen). However, this is by no means always the case. Even the presence of L and H chain labels does not always indicate a pair of light and heavy chains forming an antibody. It is therefore necessary to determine the correct pairs of light and heavy chains from the chain type assignments in the split chains. For heavy-chain-only antibodies, no antibody chain pairing needs to be performed. Light-chain-only antibodies require the two light chains (generated by symmetry if necessary) to be paired and this is done by identifying chains having the C*α* of residue L36 (light chain residue 36 using Kabat numbering) in the two chains within 20 Å of one another and the C*α* of the two L87 residues also within 20 Å. For ‘complete’ antibodies, chains are paired where there is a maximal number of contacts defined as having atom centres within 4 Å. For example, PDB file 1DVF (Figure 3) is an anti-idiotypic antibody with light chains A, C and heavy chains B, D. Light chain A makes 128 contacts with heavy chain B and 20 contacts with heavy chain D. Light chain C makes 10 contacts with heavy chain B and 117 with heavy chain D. Consequently one antibody is formed from chains A, B while the other is formed from chains C, D.

**Step 5: Assigning Antibody/Antibody Complexes.** Initially antibodies in PDB files containing no non-antibody chains (i.e. PDB files containing only light and heavy chains) are assigned as free antibodies. However, if the PDB file contains more than one antibody, it is possible that one is acting as an antigen for the other. If the sequences of the two antibodies are identical then this will not be the case, but if the sequences are different then they will be forming antibody–antigen pairs (e.g. PDB entry 1DVF, Figure 3). Thus each antibody in turn is reassigned as being a potential 2-chain antigen and processed as such in the next steps. All such antibodies are placed in the protein antigen set.

**Step 6: Assigning Free/Complexed Antibodies for HETATM Complexes.** If an antibody is currently assigned as uncomplexed, a check is now made for CDR contacts with HETATM non-protein antigens including haptens, lipids and carbohydrates which were previously removed from the PDB files. These groups are assigned as antigen if there are inter-atom contacts of <4 Å between any pairs of atom centres, but there are no CONECT records indicating that the HETATM group is covalently bound to the antibody. All such antibody complexes are placed in the non-protein antigen set.

**Step 7: Assigning Free/Complexed Antibodies for Protein and Nucleotide Antigens.** Antibodies from PDB files containing non-antibody chains (protein, DNA or RNA) are initially assumed to be complexes. However, the correct antigen chain must be identified and, in some cases, while the non-antibody protein is indeed contacting the antibody, it is not interacting with the CDRs of the antibody and should therefore be classified as an antibody-binding protein rather than as an antigen as described for PDB file 1DEE. A non-antibody chain is identified as antigen based on the following conditions: (i) more contacts are made with CDRs than with framework and (ii) at least 15 contacts are made with the CDRs. Again, contacts are defined as a distance of ≤4Å between any pair of atom centres. Antibodies can bind across antigens which have multiple chains. For example, in 4XI5, the antibody binds to chains L and H from the envelope glycoprotein of *Varicella zoster* virus (22).

**Step 8: Annotating the PDB files.** The bulk of the standard PDB header is removed and replaced by customized REMARK 950 records (not used by the wwPDB in PDB files). During numbering of the antibody chains, the chain labels are replaced by L and H as appropriate. A REMARK 950 record is used to indicate the mapping of the L and H chains to their original chain labels in the source PDB file. Antigen chains will retain their original chain label unless an antigen had the original label L or H, in which case it will be replaced by lowercase l or h, respectively. Other key annotations provided in the REMARK 950 records include: the numbering scheme applied to the antibody structure; the method by which the structure was solved; the resolution, R-factor, and R-Free where appropriate; and the antigen and antibody molecule name and species.

Antibodies binding to DNA or RNA antigen chains are added to the non-protein antigen set while others are placed in the protein antigen set. Note that some of the PDB files contain both free and complexed antibodies. The above procedure handles this, separating antibody–antigen complexes and free antibodies into their respective datasets. At this stage, the pipeline has segregated the data into three antibody types: (i) complete antibodies (heavy and light chains), (ii) light-chain-only antibodies and (iii) heavy-chain-only antibodies. Each of these types is sub-divided into three complex classes: (i) free antibodies, (ii) complexes with protein and (iii) complexes with non-protein (DNA, RNA, hapten, lipid or carbohydrate). Each of these 9 datasets is numbered with 3 numbering schemes making 27 separate datasets. In addition, another nine datasets are generated for the three antibody types and three numbering schemes, but with each set containing all three complex classes.

Thus, 36 datasets are made available, but at this stage many of the antibodies may be ‘redundant’—i.e. the same antibody appears in several files (originating from either the same PDB file or different files) and may be present both complexed and uncomplexed.

### Redundancy processing

In order to provide information about redundant antibodies, the ATOM records of the numbered antibody structures are used to extract the sequence. Each antibody pair (both light and heavy chains) is compared on the basis of the residue labels that are present in both sequences. For example, if residue L24 is present in both sequences and the amino acid is different, then the two antibodies would not be regarded as redundant. If residues are missing in one antibody compared with the other, these positions are ignored in the comparison. This process is repeated across all pairs of antibodies in order to identify redundant clusters.

In order to select a representative from each cluster, if there were differences in lengths, then the shorter sequences are discarded. From the remaining structures, the highest resolution structure is selected.

The non-redundancy processing is performed across each of the 36 (redundant) datasets described previously such that a non-redundant dataset is produced for each of the redundant sets.

In addition, lists are generated indicating the redundant clusters. A total of 12 lists are provided for each of the three antibody types (complete, light-chain-only and heavy-chain-only) and each of the four complex classes: (free, protein, non-protein, all). In addition, three more lists are provided for antibodies available both free and complexed (for each antibody type: complete, light-chain-only and heavy-chain-only).

### Implementation

The system is completely automatic and is implemented in Perl, C and Bash. The main data processing algorithm is implemented in Perl. It extracts PDB codes from the SACS XML file, does all the processing [including calling out to *Abnum* (13) to perform the numbering] and generates separate directories for each dataset of antibody structures. In-house programs to identify antibody chains (*idabchain*) and haptens (*hashapten*) and the *Abnum* numbering program are written in C. The web page is generated automatically to provide access to the data. Search options are implemented using Perl CGI scripts.

## Results

### The web interface

The data are available via a web interface at www.bioinf.org.uk/abs/abdb/. RESTful web services access to the datafiles is also provided via the web site.

#### Database searching

The resource may be searched by PDB code, keyword (antibody or antigen name) and species (of antibody or antigen). The results are presented as a table with all the antibodies that match the search query along with any antibodies that are redundant with these antibodies. The information in the table includes a downloadable antibody structure file for each of the three numbering schemes (Kabat, Chothia and Martin), the antibody status (free or complexed), resolution and R-factor.

**Figure 4.**
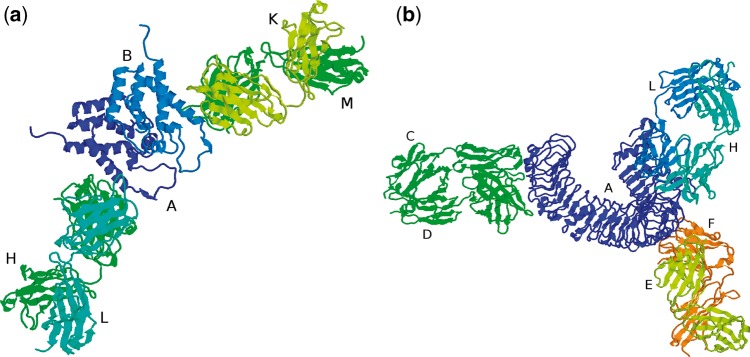
(**a**) PDB file 1AFV containing two copies of the same antibody (chains H/L and K/M) complexed with two copies of the same antigen (chains A and B), (**b**) PDB file 3ULU containing three different antibodies (chains C/D, E/F and L/H) interacting with different parts of the same antigen (chain A).

PDB files containing antibody–antigen complexes can contain multiple antibody structures (which may or may not be identical sequences) and the main processing pipeline will have split these into separate files. For example, 1AFV contains two copies of the same antibody, FAB25.3, bound to two copies of the human immunodeficiency virus type 1 capsid protein (Figure 4a). The main processing pipeline identifies chains L and H as one antibody, chains K and M as a second antibody and identifies chain A as the antigen associated with the first antibody and chain B as associated with the second antibody. The *Fv* region of the first antibody (chains L, H) together with its cognate antigen (chain A) is stored as as 1AFV_1 while the *Fv* of the second antibody and its antigen (chains K, M, B) is stored as 1AFV_2. While 1AFV only contains ‘internal redundancy’ (i.e. there are two copies of FAB25.3 in the original PDB file, but no other files contain a structure for FAB25.3), some antibodies are present in multiple PDB files. For example, a search for PDB 4KKC will display the two copies (4KKC_1 and 4KKC_2) that are present in that PDB file together with an additional 72 PDB entries having the same antibody sequence.

On the other hand, 3ULU contains three different antibodies (FAB15, FAB12 and FAB1068) bound to different parts of the same antigen (human toll-like receptor 3—chain A in the PDB file) as shown in Figure 4b. Thus the main processing pipeline generates three files, each containing a copy of the antigen and one of the antibodies: 3ULU_1 (containing chains L, H and A), 3ULU_2 (chains C, D and A) and 3ULU_3 (chains E, F and A). In such cases, redundant antibody information is provided for each of the distinct antibodies (Table 1).

**Table 1.** Non-redundant antibodies in PDB file 3ULU along with other redundant antibody structures

Query PDB	Query PDB status	Redundant PDB	Redundant PDB status
3ULU_1	Protein complex	3ULV_1	Protein complex
		3NA9_1	Non-protein complex
3ULU_2	Protein complex	3ULV_2	Protein complex
		3ULS_1	Free antibody
		3ULS_2	Free antibody
3ULU_3	Protein complex	3ULV_3	Protein complex
		3QPQ_1	Non-protein complex
		3QPQ_2	Non-protein complex
		3QPQ_3	Non-protein complex
		3QPQ_4	Non-protein complex

In addition to the search by PDB code, the database can be queried by a keyword from the antibody or antigen name. For example, all the structures for the HyHEL antibodies (anti-hen egg white lysozyme) from the Smith-Gill group (23) can be queried using ‘hyhel’. Similarly, all the capsid-antigen-bound antibodies can be queried by searching for ‘capsid’. Data can also be searched by antibody and antigen species and a drop-down menu has been provided with all the species of antibody and antigen observed in the dataset.

#### Data download

As well as individual PDB files, a compressed archive of each of the 72 antibody structures datasets (the 36 redundant and 36 non-redundant sets described above) can be downloaded. In addition, the following information can be downloaded: (i) the 12 lists of redundancy information—one for each of the three antibody types (complete, light-chain-only and heavy-chain-only) and each of the four complex classes (free, protein, non-protein, all); (ii) three lists of antibodies (one for each type: complete, light-chain-only and heavy-chain-only) that are available both free and complexed and (iii) a list of mappings between chains for each antibody/antigen complex and the chain identifiers in the original PDB files for people wishing to obtain the full Fab fragments from the original files.

### Database statistics

A list of 2918 antibody structure PDB codes was obtained from SACS. Of these, 2542 PDB files were successfully processed to generate 4300 PDB files: 3731 complete antibodies, 144 light-chain-only and 425 heavy-chain-only. A detailed breakdown of the content of the AbDb database as of October 2017 is shown in Table 2.

**Table 2.** Contents of the AbDb datasets, October 2017

Datasets	Complex type	Processed PDB files	Resultant antibodies	Non-redundant antibodies
Complete antibody	Protein	1225	2067	866
	Non-protein	353	493	244
	Free antibody	688	1171	623
	Complete dataset	2228	3731	1476
Light chains	Protein	6	7	4
	Non-protein	14	20	7
	Light only	76	117	50
	Complete dataset	96	144	57
Heavy chains	Protein	149	280	118
	Non-protein	9	16	9
	Heavy only	68	129	66
	Complete dataset	218	425	177

The SACS database which provides lists of antibodies to be processed by AbDb is a cumulative resource (listing PDB files that have now been obsoleted) that also contains information on structures of antibody *Fc* fragments. Consequently there are a number of files that are not processed by AbDb: 179 *Fc* fragments and 4 obsoleted. In addition, the numbering procedure fails for at least 1 of the chains in 119 PDB files and 65 files are identified as containing single chain *Fv*s (*scFv*s) with a single chain label for the heavy- and light-chain-derived parts of the sequence. *scFv*s are fused V_H_ (variable heavy) and V_L_ (variable light) domains with a peptide linker (24). Frequently the peptide linker is flexible and is not visible in the PDB file and, in these cases, while part of a single chain, the PDB file often has different chain labels for the V_H_ and V_L_ regions. Such examples are processed correctly by AbDb. However, in cases where the two regions have been given the same chain label, the *Abnum* program is currently not able to separate the two regions and therefore does not number the protein correctly. Consequently these files are currently automatically rejected.

## Discussion

AbDb provides a valuable resource for the structural immunologist, collecting pre-numbered structures of *Fv* fragments together with their cognate antigens where available. We have used this resource for a number of applications including assessment of antibody modelling, analysis of the variation in loop conformation on antigen binding and analysis of epitope conformation (Ferdous and Martin, in preparation). An earlier version was used for the analysis of the packing between the two variable domains (V_H_ and V_L_) (25).

AbDb provides a regularly updated resource with processed antibody structures and has a number of features not provided by other antibody structure resources. In particular, it provides:

- Processed PDB files containing only the variable domains and split into individual antibodies with cognate antigens (including multi-chain and non-protein antigens),
- PDB files numbered with Kabat, Chothia and Martin numbering schemes,
- A total of 36 simply classified downloadable datasets: complete antibodies, light-chain-only and heavy-chain-only, also split into free antibodies, complexes with protein antigens and complexes with non-protein antigens (all numbered with all three numbering schemes),
- Non-redundant versions of each of the 36 datasets,
- A total of 12 information files describing redundant clusters,
- Information on redundancy when searching by PDB code: a list of all processed PDB files containing redundant antibodies is provided,
- Three information files describing antibodies available both complexed and uncomplexed,
- Non-antibody proteins, not interacting predominantly with the CDRs, are not treated as antigen and the antibody is classified as uncomplexed (e.g. 1DEE),
- Antibodies involved in anti-idiotype interactions are classified both as antibodies and antigens,
- Light-chain dimers regenerated by exploiting symmetry information from the PDB file.

Unfortunately it is almost impossible to predict every variant of antibody structures that may become available in the future. Consequently AbDb is continuously being updated to handle unforeseen circumstances as they are encountered. The desire is to make the dataset clean and robust at the possible expense of missing some of the available structures. Future planned enhancements include improved processing of *scFv*s (single chain variable fragments) and single chain *Fab*s (*scFab*s) where a single chain label is used for light and heavy chain segments in the PDB file. Currently, our *idabchain* program which identifies chain types identifies these as hybrid chains, but the AbDb pipeline does not process them further owing to current restrictions in the numbering program *Abnum*. We therefore intend to improve *Abnum* to deal correctly with these examples. While we have not yet encountered any structures, antibody drugs have been created having *scFv*s fused to the C-terminus of a conventional heavy chain; AbDb would currently ignore the *scFv* fusion and we need to create a general method for dealing with fusions. *Abnum* has been designed to fail cleanly with unusual structures rather than provide incorrect numbering, but we plan to enhance its ability to number these unusual cases. Similarly, the symmetry information provided in the PDB file has only been exploited for light-chain-only antibodies, but in future will be expanded to handle all structures to ensure that we use biological assemblies.

The PDB file format is officially deprecated, but still widely used in structural bioinformatics. Future versions of AbDb will add support for the newer mmCIF and PDBML format files. In addition, the wwPDB has recently announced that the current four-character PDB code will be replaced by an eight-character PDB identifier together with a version number. AbDb will need to be modified to support this new format. Plans to enhance the web server include the addition of an integrated graphical viewer and additional RESTful access to the data.
